# 播散肿瘤细胞免疫逃逸机制及其在肿瘤转移中的作用研究进展

**DOI:** 10.3779/j.issn.1009-3419.2025.101.23

**Published:** 2025-12-20

**Authors:** Zujun QUE, Bin LUO, Jianhui TIAN

**Affiliations:** 201801 上海，上海中医药大学附属市中医医院肿瘤临床医学中心，肿瘤研究所; Institute of Oncology, Clinical Oncology Center, Shanghai Municipal Hospital of Traditional Chinese Medicine, Shanghai University of Traditional Chinese Medicine, Shanghai 201801, China

**Keywords:** 肿瘤转移, 播散肿瘤细胞, 免疫逃逸, 正虚伏毒, 转移态, Tumor metastasis, Disseminated tumor cells, Immune evasion, Hidden toxicity due to vital qi deficiency, Metastatic state

## Abstract

肿瘤转移是导致癌症患者死亡的首要原因，而播散肿瘤细胞（disseminated tumor cells, DTCs）作为转移级联反应中的关键“种子”，其命运直接决定了转移的成败。DTCs从原发灶脱落并定殖于远处靶器官后，常进入一种长期休眠状态，随后通过复杂的免疫逃逸机制突破免疫监视，从休眠态激活为增殖态，最终形成临床可评估的转移灶。深入理解DTCs的免疫逃逸机制对于揭示肿瘤转移的本质和开发有效的抗转移策略至关重要。本文系统综述了DTCs免疫逃逸机制的最新研究进展，重点围绕三大核心机制展开：抗原呈递缺陷、免疫抑制微环境的形成以及代谢重编程介导的免疫抑制。具体而言，DTCs通过下调主要组织相容性复合体I类（major histocompatibility complex class-I, MHC-I）分子表达实现“免疫隐身”；主动招募调节性T细胞（regulatory T cells, Tregs）、髓源性抑制细胞（myeloid-derived suppressor cells, MDSCs）等免疫抑制性细胞，构建局部“防护盾”；并通过重塑糖、氨基酸及脂质代谢，从能量与物质层面削弱效应免疫细胞功能。在此基础上，本文创新性地结合中医“正虚伏毒”与“转移态”理论，阐释机体正气盛衰与DTCs休眠-觉醒转换之间的动态病机关系，为从整体调控角度认识转移过程提供了新视角。最后，文章展望了针对DTCs免疫逃逸的多靶点联合治疗策略，以及单细胞测序、空间转录组等新技术在该领域的应用前景，以期为未来抗转移研究的深入与临床转化提供重要参考。

## Abstract (en)

Tumor metastasis is the leading cause of cancer-related mortality. Disseminated tumor cells (DTCs), serving as the critical ‘seeds’ in the metastatic cascade, hold the key to determining the success or failure of metastasis. Following dissemination from the primary tumor and colonization of distant organs, DTCs often enter a prolonged state of dormancy. Their subsequent escape from immune surveillance through sophisticated mechanisms enables them to transition from this dormant state to a proliferative one, ultimately culminating in clinically detectable metastatic lesions. A profound understanding of DTCs immune evasion is therefore essential for unraveling the fundamental biology of metastasis and developing effective anti-metastatic strategies. This article systematically reviewed the latest advances in the mechanisms underlying DTCs immune evasion, focusing on three core aspects: defects in antigen presentation, formation of an immunosuppressive microenvironment, and metabolism reprogramming-mediated immunosuppression. Specifically, DTCs achieve ‘immune invisibility’ by downregulating major histocompatibility complex class-I (MHC-I) molecule expression; they actively construct a local ‘protective shield’ by recruiting immunosuppressive cells such as regulatory T cells (Tregs) and myeloid-derived suppressor cells (MDSCs); and they impair effector immune cell function at the energetic and metabolic levels by remodeling glucose, amino acid, and lipid metabolism. Building upon this, we innovatively integrate the traditional Chinese medicine (TCM) theories of ‘hidden toxicity due to vital qi deficiency’ and the ‘metastatic state’ to elucidate the dynamic pathogenic relationship between the body’s systemic ‘Zhengqi’ (vital energy) status and the dormancy-awakening switch of DTCs, offering a novel holistic perspective for comprehending the metastatic process. Finally, we discussed the prospects of multi-target combination therapeutic strategies against DTCs immune evasion and highlight the potential of emerging technologies, such as single-cell sequencing and spatial transcriptomics, aiming to provide valuable insights for future in-depth research and clinical translation in the field of anti-metastasis therapy.

肿瘤转移是造成癌症患者死亡的主要原因，约占癌症相关死亡的90%^[1,2]^。尽管现代医学在手术、化疗、分子靶向治疗及免疫治疗等领域取得了进展，但肿瘤转移的防治仍是临床瓶颈^[1]^。即使早期肺癌患者采取根治性手术，术后接受辅助治疗，但转移率仍高达40%，5年生存率仅约60%^[2]^。在肿瘤转移这一复杂过程中，播散肿瘤细胞（disseminated tumor cells, DTCs）作为转移的“种子”，其免疫逃逸能力是决定转移成败的关键因素之一。近年来，随着单细胞测序技术和微环境分析方法的进步，DTCs的免疫逃逸机制研究取得了显著进展，深入理解这些机制不仅有助于阐明肿瘤转移的生物学本质，也为开发新型抗转移治疗策略提供理论依据。本文系统综述DTCs免疫逃逸机制的最新研究进展，以期为临床转化研究提供新思路。

## 1 DTCs免疫逃逸与临床肿瘤转移

DTCs免疫逃逸是临床肿瘤转移发生的最终一环。DTCs是指从原发灶脱落并播散至远处靶器官的肿瘤细胞群体。在肿瘤转移级联过程中，原发肿瘤细胞突破基底膜进入外周循环系统，部分细胞存活并成为循环肿瘤细胞（circulating tumor cells, CTCs），CTCs通过循环系统进一步播散至远处组织器官形成DTCs，DTCs一旦在远处组织器官中成功定殖，可在适宜的微环境下重新激活、增殖并形成转移灶^[3]^。值得注意的是，DTCs并非立即增殖，而是常进入一种可维持数月甚至数年的休眠态，这使其能逃避常规治疗和免疫清除^[4,5]^。例如在肺腺癌转移模型^[6,7]^中，休眠态DTCs通过沉默干扰素基因刺激因子（stimulator of interferon gene, STING）信号通路，避免招募自然杀伤（natural killer, NK）细胞和T细胞，从而实现免疫逃逸。类似地，在肝癌研究^[8]^中发现，DTCs中血清和糖皮质激素诱导激酶1（serum- and glucocorticoid-inducible kinase 1, *SGK1*）的缺失可抑制CD8^+^ T细胞介导的杀伤，并通过重塑抑制性微环境促进转移。休眠态的DTCs能够在靶器官中长期存活，并有效逃避常规的抗肿瘤治疗和免疫系统的攻击。这些研究表明，DTCs在靶器官中的持久存活与其主动抑制局部免疫应答密切相关。

随着时间推移，DTCs可通过与微环境中基质细胞的相互作用，从休眠态向增殖态转换。例如在黑色素瘤肺转移模型^[9]^中，成纤维细胞通过分泌分泌型卷曲相关蛋白1（secreted frizzled related protein 1, sFRP1）抑制WNT5A信号，从而诱导休眠态细胞再激活。化疗等外部压力也可通过诱导肺内成纤维细胞衰老和中性粒细胞胞外诱捕网（neutrophil extracellular trap, NET）形成，推动DTCs增殖^[10]^。因此，DTCs在转移靶器官中成功实现免疫逃逸，是其突破休眠、最终形成临床可检测转移灶的关键步骤。

## 2 DTCs免疫逃逸的关键机制

免疫编辑理论揭示了免疫系统与肿瘤细胞之间的动态博弈，包括清除、平衡与逃逸3个阶段。在逃逸阶段，肿瘤细胞通过多种机制逃避免疫识别与攻击，最终形成转移灶^[11]^。DTCs作为转移的种子，其免疫逃逸策略具有多层次、网络化的特点，主要涵盖以下3个方面：抗原呈递缺陷实现“免疫隐身”，构建免疫抑制微环境形成“防护盾”，以及通过代谢重编程“能量劫持”以削弱免疫细胞功能。

### 2.1 DTCs抗原呈递缺陷机制

DTCs通过下调主要组织相容性复合体I类（major histocompatibility complex class-I, MHC-I）分子表达逃避免疫识别，这一经典策略不仅使DTCs难以被免疫细胞识别，也与放化疗及免疫治疗耐药密切相关^[12]^。DTCs可通过多种表观遗传和信号通路调控机制主动抑制MHC-I表达。例如在乳腺癌脑转移中，星形胶质细胞来源的弹性蛋白微纤维界面定位蛋白1（elastin microfibril interface located protein 1, Emilin-1）可诱导DTCs中细胞周期蛋白依赖性激酶5（cyclin-dependent kinase 5, Cdk5）过表达，进而抑制信号转导和转录激活因子1（signal transducer and activator of transcription 1, STAT1）/importin α/核苷酸结合寡聚结构域（nucleotide-binding and oligomerization domain, NOD）样受体家族含CARD结构域5（NOD-like receptor family caspase recruitment domain family domain-containing 5, NLRC5）轴，导致MHC-I类分子下调，帮助DTCs逃避CD8^+^ T细胞的识别与杀伤^[13]^。核仁磷酸蛋白1（nucleophosmin 1, NPM1）则通过结合干扰素调节因子1（interferon regulatory factor 1, IRF1）阻遏其与MHC-I启动子结合，从而抑制抗原呈递^[14]^。表观遗传调控也起关键作用：多梳抑制复合物2（polycomb repressive complex 2, PRC2）可通过维持*MHC-I*基因启动子的H3K27me3修饰沉默其表达^[15]^，而抑制Zeste同源物2增强子（enhancer of zeste homolog 2, EZH2）或脂肪酸合成酶（fatty acid synthase, FASN）则可分别通过降低H3K27me3水平或减少MHC-I棕榈酰化降解，从而增强抗原呈递与T细胞杀伤^[16]^。此外，抗原加工与呈递相关基因的表达异常也会削弱DTCs的免疫原性，例如在乳腺癌^[17]^中，孕酮受体通过孕激素信号下调抗原加工与呈递相关基因及MHC-I类分子表达，进而减少CD8^+^ T细胞介导的杀伤；E3泛素连接酶*Parkin*缺失可通过调控磷酸酶及张力蛋白同源基因（phosphatase and tensin homolog, PTEN）/磷脂酰肌醇3-激酶（phosphatidylinositol 3-kinase, PI3K）/蛋白激酶B（protein kinase B, AKT）通路降低抗原提呈能力，导致CD8^+^ T细胞浸润与活化不足^[18]^；树突状细胞（dendritic cells, DCs）中m^6^A结合蛋白YTHDF1可通过加速抗原降解抑制交叉提呈，从而削弱CD8^+^ T细胞活化^[19]^。相反，人工激活cGAS-STING通路^[20]^、抑制BCL9/BCL9L^[21]^或多配体蛋白聚糖-1（syndecan-1, SDC1）^[22]^均可增强抗原呈递与T细胞应答，而抑制RNF31则可通过“旁观者杀伤”机制对抗原呈递缺陷的肿瘤细胞产生广泛免疫清除作用^[23]^。抗原呈递缺陷并非DTCs免疫逃逸的唯一机制，研究^[24]^提示休眠DTCs的逃逸可能更多源于其与T细胞相遇的“空间稀缺性”，增加T细胞数量（如过继性细胞免疫治疗）可有效清除休眠DTCs，表明DTCs免疫逃逸是一个多因素、动态适应的过程，涉及抗原呈递缺陷与局部免疫抑制的双重保障。综上所述，DTCs通过下调抗原呈递相关分子实现“免疫隐身”，从而逃避T细胞的直接识别与杀伤；而除了这种被动策略，DTCs还通过主动塑造免疫抑制微环境构建“防护盾”，形成其实现免疫逃逸的双重核心机制。

### 2.2 免疫抑制微环境形成

在实现“隐身”的同时，DTCs还主动塑造免疫抑制微环境，通过分泌趋化因子与细胞因子[如C-C基序趋化因子配体5（C-C motif chemokine ligand 5, CCL5）、C-X-C趋化因子配体12（C-X-C motif chemokine ligand 2, CXCL12）、转化生长因子-β（transforming growth factor-β, TGF-β）、白细胞介素-10（interleukin-10, IL-10）等]，系统性招募并活化多种抑制性免疫细胞，形成局部免疫屏障。这些细胞包括调节性T细胞（regulatory T cells, Tregs）、髓源性抑制细胞（myeloid-derived suppressor cells, MDSCs）、M2型肿瘤相关巨噬细胞（tumor-associated macrophages, TAMs）以及肿瘤相关中性粒细胞（tumor-associated neutrophils, TANs）等，它们通过分泌抑制性因子或直接细胞接触，协同抑制效应免疫细胞功能^[25]^。其来源、核心免疫抑制功能及关键效应分子见表1^[26-30]^。

**表 1:** 主要免疫抑制细胞在DTCs微环境中的作用及其效应分子

Immunosuppressive cell type	Source	Core immunosuppressive functions	Key effector molecules
Tregs^[26]^	Recruitment from peripheral circulation, local induction/differentiation	Suppress activation/proliferation of CD4^+^/CD8^+^ T cells, impair NK cell cytotoxicity	IL-10, TGF-β, CTLA-4, PD-1
MDSCs^[27]^	Expansion of immature myeloid cells in bone marrow	Deplete arginine/tryptophan, produce ROS/iNOS, induce T cell exhaustion	ARG1, iNOS, ROS, CXCL5
M2-TAMs^[28,29]^	Recruitment of peripheral monocytes, local macrophage polarization	Inhibit effector T cell infiltration, promote angiogenesis, remodel extracellular matrix	IL-10, TGF-β, VEGF, CCL5
TANs^[30]^	Bone marrow mobilization, local recruitment	Form NETs to capture DTCs, suppress T cell function, promote DTCs proliferation	IL-8, IL-1β, NETs, CXCL2

DTCs: disseminated tumor cells; Tregs: regulatory T cells; MDSCs: myeloid-derived suppressor cells; TAMs: tumor-associated macrophages; TANs: tumor-associated neutrophils; IL: interleukin; TGF-β: transforming growth factor-β; CTLA-4: cytotoxic T-lymphocyte-associated protein 4; PD-1: programmed cell death protein 1; ARG1: arginase 1; iNOS: inducible nitric oxide synthase; ROS: reactive oxygen species; CXCL5: C-X-C motif chemokine ligand 5; VEGF: vascular endothelial growth factor; CCL5: C-C motif chemokine ligand 5; CXCL2: C-X-C motif chemokine ligand 2; NETs: neutrophil extracellular traps; NK: natural killer.

Treg通过分泌IL-10、TGF-β等抑制性细胞因子^[26,31]^，以及经SPP1^[32]^、TGF-β/Smad^[33]^、钙网蛋白（calreticulin, CALR）/Janus激酶（Janus kinase, JAK）/PI3K^[34]^等信号通路介导的主动招募，显著抑制CD4^+^/CD8^+^ T细胞的活化和增殖。MDSCs则通过精氨酸酶1（arginase 1, ARG1）、诱导型一氧化氮合酶（inducible nitric oxide synthase, iNOS）及活性氧（reactive oxygen species, ROS）等介质，耗竭微环境中的精氨酸并促进Tregs分化，协同增强免疫抑制^[27]^，其扩增与活化受CXCL5、粒细胞-巨噬细胞集落刺激因子（granulocyte-macrophage colony-stimulating factor, GM-CSF）^[35]^、PGLYRP1/肿瘤坏死因子-α（tumor necrosis factor-α, TNF-α）^[36]^及G9a/Fbxw7/Notch^[37]^等信号通路的调控。M2-TAMs在IL-6/JAK2/STAT3、外泌体介导的缺氧诱导因子1α（hypoxia-inducible factor 1α, HIF1α）/LDHA/STAT3及Notch/c-MYC等信号通路驱动下极化为免疫抑制表型，通过分泌血管内皮生长因子（vascular endothelial growth factor, VEGF）、IL-10、TGF-β等促进血管生成、组织重塑及效应T细胞功能抑制^[28,38]^。TANs则通过形成NET及分泌IL-8、IL-1β等因子，在富组氨酸糖蛋白（histidine-rich glycoprotein, HRG）/FcγRI/PI3K/核因子-κB（nuclear factor-κB, NF-κB）^[39]^、STAT6-C3^[40]^、CTSC^[41]^、CXCL2/CCL4/STAT3^[42]^、FGF19/FGFR4/JAK2/STAT3^[43]^及SETD2/CXADR/PI3K/AKT^[44]^等信号通路的调控下，直接捕获并激活DTCs，同时抑制T细胞功能。此外，基质细胞（如间充质干细胞、破骨细胞、肝星状细胞等）也通过ER/SCUBE2/SHH、胶原蛋白/LAIR1^[45]^、Siglec15/TLR2^[46]^及GM-CSF/GM-CSFR^[47]^等信号通路参与免疫抑制微环境的构建。综上，DTCs通过多重细胞与信号通路网络协同构建免疫抑制微环境，为其在远处器官中的定殖、休眠突破及转移灶形成提供关键支持。

### 2.3 代谢重编程介导的免疫抑制

除了细胞层面的调控，DTCs还可通过代谢重编程改变微环境代谢格局，从能量和物质层面抑制免疫细胞功能。在糖代谢方面，肿瘤细胞通常增强有氧糖酵解（Warburg效应），大量摄取葡萄糖并产生乳酸，导致微环境中葡萄糖匮乏和酸化，直接抑制CD8^+^ T细胞活力和NK细胞毒性，并促进M2-TAMs极化^[48]^。有研究^[49]^揭示，肿瘤内α-酮戊二酸依赖性双加氧酶（alpha-ketoglutarate dependent dioxygenase, FTO）通过去甲基化修饰增强糖酵解基因表达，加剧葡萄糖竞争，形成代谢屏障。乳腺癌干细胞则通过分泌巨噬细胞移动抑制因子（macrophage migration inhibitory factor, MIF）激活Wnt/β-catenin/c-MYC信号轴，上调醛缩酶C（aldolase C, ALDOC）的表达，驱动糖酵解重编程与免疫抑制^[50]^。氨基酸代谢异常也是重要机制。色氨酸代谢关键酶——色氨酸2,3-双加氧酶（tryptophan 2,3-dioxygenase, TDO2）在转移灶成纤维细胞中高表达，将色氨酸转化为犬尿氨酸（kynurenine, KYN），后者通过激活芳香烃受体（aryl hydrocarbon receptor, AHR）诱导T细胞耗竭，并上调程序性死亡配体1（programmed death-ligand 1, PD-L1）表达，支持DTCs的免疫逃逸^[51]^。MDSCs高表达的ARG1可大量消耗微环境中的精氨酸，导致T细胞因精氨酸匮乏而出现细胞周期阻滞和功能受损，从而无法有效清除肿瘤细胞^[27]^。此外，脂质代谢的重编程同样参与调控免疫微环境。在乳腺癌肺转移前微环境中，肺间充质细胞通过前列腺素E2-E型前列腺素受体2（prostaglandin E2-E-prostanoid receptor 2, PGE2-EP2）信号抑制中性粒细胞脂解，促进脂质积聚并被肿瘤细胞摄取，增强其氧化磷酸化能力，最终促进肺转移灶的形成^[52]^。此外，乳酸不仅作为代谢产物，还可通过组蛋白乳酸化修饰上调CD47和激活STAT3，进一步促进免疫逃逸^[53]^。由此可见，DTCs通过糖、氨基酸和脂质代谢的重编程，构建了一个“代谢-免疫”协同抑制网络，为其在远处器官中的定殖与增殖提供多重保障。

## 3 中医对DTCs免疫逃逸及转移发生的病机认识

田建辉教授^[54]^创建的中医肿瘤转移“正虚伏毒”病机理论和肿瘤“转移态”学说，为理解DTCs的免疫逃逸及转移灶形成提供了独特的视角和病机框架。在该理论中，作为转移“种子”的CTCs及DTCs，其特性与行为（如长期潜伏、伺机而动、危害性强）可对应于中医学的“伏毒”；而机体的全身免疫状态及局部微环境稳态，则体现了“正气”的盛衰功能。“伏毒”具有“伏于血道，藏于脏腑，流注全身，正盛则毒伏而不出，正虚则毒出而为病”的致病特点。这一认识精准地概括了DTCs增殖休眠与临床转移发生之间的动态演变过程：当人体正气充盛（即免疫系统功能健全、微环境处于稳态）时，DTCs得以被抑制并长期处于休眠状态（“正盛毒伏”），不形成影像学可见的转移灶；而当正气亏虚（表现为免疫监视功能下降、免疫抑制性微环境形成、代谢失衡等微观层面的“内环境失和”）时，DTCs则从休眠状态转变为增殖状态（“正虚毒发”），最终进展为临床可检测的转移灶^[55]^。现代免疫学研究进一步从细胞与分子层面为此提供了证据：CD39^+^程序性死亡受体1（programmed cell death 1, PD-1）^+^CD8^+^ T细胞^[56]^、NK细胞^[57]^等功能性免疫细胞可维持DTCs的休眠状态，可视为“正气”在特定微环境中的体现；而其功能障碍或数量减少（“正气亏虚”）时，DTCs便得以突破免疫约束，通过前述的抗原下调、招募抑制性细胞、代谢重编程等机制（“毒发”的具体病理环节）实现免疫逃逸，进入增殖并形成转移灶。这与中医“正虚毒发”的病机高度吻合。因此，在防治策略上，维持“正气充盛、毒邪隐伏”的动态平衡，防止“正气亏虚、伏毒复发”，是阻断转移灶形成的关键环节^[54]^。

从分子机制层面看，“正虚”则对应机体免疫防御功能在分子层面的多重缺陷，具体表现为CD8^+^ T细胞浸润不足，TNF-α、干扰素-γ（interferon-γ, IFN-γ）、颗粒酶B和穿孔素分泌下降，同时伴有Tregs、MDSCs等免疫抑制性细胞富集，以及IL-10、TGF-β等抑制性细胞因子高表达，共同构成了局部“正虚”的微观病理状态。“伏毒”——休眠态DTC呈现高表达p27、p21等细胞周期抑制蛋白，低表达Ki-67等增殖标志物，并通过下调NKG2D配体、沉默STING信号通路等方式实现免疫逃逸。值得关注的是，中医“扶正”治法相关药物（如黄芪、党参）的现代药理学研究进一步揭示，其活性成分可通过激活CD8^+^ T细胞功能^[58]^、抑制M2-TAMs极化^[59]^、下调PD-L1表达^[60]^等机制增强机体抗肿瘤免疫应答，从而在分子层面为“正虚伏毒”理论提供了实验依据，也为干预DTCs休眠-再激活转化及防治肿瘤转移提供了潜在的中西医结合调控靶点。中医药通过整体调节以增强正气、稳定内环境，可能为抑制DTCs活化、延缓转移发生提供重要的治疗思路。中医“正虚伏毒”理论为解读DTCs免疫逃逸的复杂机制提供了整体视角，而现代生物学机制解析则为靶向干预提供了具体靶点，二者结合为抗转移策略的开发提供了新思路。

## 4 总结与展望

DTCs的免疫逃逸是肿瘤转移形成的核心环节，其机制涵盖抗原呈递异常、免疫抑制微环境的主动塑造以及代谢重编程介导的免疫调控等多个相互关联的层面。这些机制并非孤立存在，而是协同作用：抗原呈递缺陷使DTCs实现“免疫隐身”，免疫抑制微环境的构建为其提供局部“防护盾”，而代谢重编程则从能量和物质基础层面削弱免疫细胞功能，共同促使DTCs在远处器官中存活并最终形成转移灶。值得注意的是，微环境中的免疫压力是诱导和维持DTCs休眠的关键因素之一，而免疫逃逸机制的启动则与DTCs从休眠到觉醒的转换密切相关。当前，针对DTCs的干预策略已初见雏形，例如通过表观遗传药物恢复MHC-I类分子表达、靶向特定趋化因子或细胞因子受体调控免疫抑制微环境或通过代谢酶抑制剂逆转免疫代谢抑制等。这些进展不仅提示了靶向DTCs的治疗潜力，也凸显了开发早期检测与精准干预方法的迫切性。

未来研究需进一步聚焦于不同转移靶器官中DTCs免疫逃逸的器官特异性。DTCs在骨、脑、肝、肺等不同器官中面临的微环境压力与逃逸策略存在显著差异：在骨转移中，DTCs通过激活破骨细胞、释放TGF-β并招募MDSCs与Tregs细胞，同时借助Siglec15等分子直接抑制CD8^+^ T细胞功能；在脑转移中，DTCs主要依赖诱导小胶质细胞向M2表型极化，并高表达PD-L1与CD47以适配“免疫特许”环境；在肝转移中，DTCs通过FGF19/FGFR4/JAK2/STAT3等通路激活肝星状细胞，促进炎症性成纤维细胞转化与NET形成；在肺转移中，则侧重于招募Tregs与M2-TAMs、促进NET形成并通过糖酵解竞争抑制CD8^+ ^T细胞功能。阐明这些器官特异性机制，为开发部位导向的个性化抗转移策略提供了关键依据。展望未来，多靶点联合治疗（如免疫检查点抑制剂与代谢调节剂联用、靶向休眠DTCs的药物协同免疫治疗、中医药整体调节联合靶向治疗等）有望通过多机制协同克服DTCs的免疫逃逸。新技术的应用将成为关键推动力：单细胞测序、空间转录组学、荧光双光子显微技术、类器官模型等工具有助于对DTCs异质性、微环境互作及免疫逃逸时空动态进行深入解析，加速临床转化进程。通过多学科交叉与融合，有望在DTCs研究与抗转移治疗领域实现重要突破，最终改善肿瘤患者的长期预后和生活质量。
